# Linking ecosystem services, urban form and green space configuration using multivariate landscape metric analysis

**DOI:** 10.1007/s10980-018-0618-z

**Published:** 2018-02-19

**Authors:** Darren R. Grafius, Ron Corstanje, Jim A. Harris

**Affiliations:** School of Water, Energy and Environment, Cranfield University, Cranfield, MK43 0AL Bedfordshire UK

**Keywords:** Landscape metrics, Fragstats, Urban, Landscape structure, Ecosystem services, Multivariate, United Kingdom

## Abstract

**Context:**

Landscape metrics represent powerful tools for quantifying landscape structure, but uncertainties persist around their interpretation. Urban settings add unique considerations, containing habitat structures driven by the surrounding built-up environment. Understanding urban ecosystems, however, should focus on the habitats rather than the matrix.

**Objectives:**

We coupled a multivariate approach with landscape metric analysis to overcome existing shortcomings in interpretation. We then explored relationships between landscape characteristics and modelled ecosystem service provision.

**Methods:**

We used principal component analysis and cluster analysis to isolate the most effective measures of landscape variability and then grouped habitat patches according to their attributes, independent of the surrounding urban form. We compared results to the modelled provision of three ecosystem services. Seven classes resulting from cluster analysis were separated primarily on patch area, and secondarily by measures of shape complexity and inter-patch distance.

**Results:**

When compared to modelled ecosystem services, larger patches up to 10 ha in size consistently stored more carbon per area and supported more pollinators, while exhibiting a greater risk of soil erosion. Smaller, isolated patches showed the opposite, and patches larger than 10 ha exhibited no additional areal benefit.

**Conclusions:**

Multivariate landscape metric analysis offers greater confidence and consistency than analysing landscape metrics individually. Independent classification avoids the influence of the urban matrix surrounding habitats of interest, and allows patches to be grouped according to their own attributes. Such a grouping is useful as it may correlate more strongly with the characteristics of landscape structure that directly affect ecosystem function.

**Electronic supplementary material:**

The online version of this article (10.1007/s10980-018-0618-z) contains supplementary material, which is available to authorized users.

## Introduction

In many cities, the provision of green space can play a significant role in maximising the benefits and minimising the negative effects of urban living, and these natural components of urban areas can provide ecosystem services in situ, such as carbon sequestration, flood mitigation, aesthetic pleasure and pollination (Szlavecz et al. 2011). Ecosystem services can be considered as outcomes of environmental processes which are then utilised by humans (Millennium Ecosystem Assessment 2005). Providing adequate green infrastructure, and therefore the ecosystem services which flow from it, can be effective solutions for both climate change adaptation and mitigation in cities (Elmqvist et al. 2015). Chiesura (2004) identified the crucial role that urban parks play in securing sustainability, through direct biophysical links to health and to psychological well-being through exposure to nature (e.g., Shanahan et al. 2015; Soga et al. 2015; Cox et al. 2017b), however exposure to nature may be limited in urban populations due to lack of easy access to green infrastructure (Cox et al. 2017a). A recent systematic review by van den Berg et al. (2015) demonstrated strong evidence for significant positive associations between the quantity of green space and perceived mental health and reduced mortality, and moderate evidence for an association with perceived general health. Sirakaya et al. (2017) have called for legal protection, restoration and investment in green spaces in urban areas. In order to do this it is essential that urban planners be provided with reliable metrics as to the size, distribution and composition of green spaces required to secure adequate ecosystem service provision if fine grained decision making at the “street” level are to be made (Grêt-Regamey et al. 2015).

Numerous recent studies have worked to determine relationships between ecosystem health and metrics of landscape structure such as patch size and complexity (Stefanov and Netzband 2005; Tratalos et al. 2007; Norton et al. 2016). The ability to quantify and analyse the spatial configuration of a landscape through the calculation and analysis of landscape metrics has enabled powerful new avenues of research, while also generating extensive discussion and disagreement around the interpretation and usefulness of these metrics (Neel et al. 2004; Wang and Malanson 2007; Cushman et al. 2008; Kupfer 2012). In natural landscapes it is often unclear exactly what impact is had on ecological function by measureable variables on the size, shape and structure of habitat patches in the landscape. In urban settings these relationships can be even less clear due to the landscape’s intense complexity and heterogeneity at fine scales, and difficulty determining which features do and do not constitute ‘habitat’ (Zhu et al. 2006; LaPoint et al. 2015; Grafius et al. 2016).

The complexity of the urban landscape increases the difficulty involved in understanding and modelling relationships between landscape structure and ecological function (Alberti 2005; Holt et al. 2015). Relying on broad, aggregate methods and metrics for conceptualising the ecological significance of urban characteristics (e.g., distance to city centre, land use or percentage cover of impermeable surfaces) risks oversimplifying the relationships between urban ecosystem function and landscape structure. Therefore there is a need to move past these relatively ‘easy’ approaches and seek novel methods that account for the unique characteristics and scale demands of the urban environment (McDonnell and Hahs 2013; Norton et al. 2016; McDonnell and MacGregor-Fors 2016). Generalisations and common principles can nonetheless be carried over from approaches used in more natural settings; for example, species-area relationships have been found to be equally valid in urban green spaces as in natural habitats despite the manipulated nature of urban environments (Ferenc et al. 2014; Nielsen et al. 2014). Challenges emerge from determining which principles and methods do not transfer from rural to urban versus which do, and how these must be adapted to validly consider the unique nature of the urban landscape.

Many studies considering urban form (i.e., the patterns and configuration associated with different land uses, histories, etc.) focus on the character and purpose of built infrastructure (e.g., Van de Voorde et al. 2011; Voltersen et al. 2014; Hecht et al. 2015). However, how this urban form affects and defines the character of urban green spaces within these areas is less understood. Urban green spaces are often categorised and analysed according to how they are used by human society and the character of the built-up land that surrounds them (Uy and Nakagoshi 2007; Park et al. 2014; Lu 2015). While this remains a relatively straightforward way to categorise urban green spaces, a more complete ecological understanding must focus on the habitats themselves rather than the matrix containing them.

The purpose of this research was to analyse the spatial form and characteristics of urban green space in the urban landscape, not only in relation to different broad classes of surrounding urban form but also independently of them. This was done through the use of landscape metrics for the towns of Milton Keynes, Bedford and Luton, UK. Generalised relationships between urban form classes and landscape metrics were initially studied as a baseline; a multivariate analysis was then conducted on calculated metrics to identify the main causes of variability among landscape patches without the biases potentially introduced by preconceived classifications of urban form. Lastly, three modelled ecosystem services (described in Grafius et al. 2016) were compared with urban forms and the multivariate patch classification to discern relationships between urban green space form and potential ecosystem service provision.

## Methods

### Study area

**Fig. 1:**
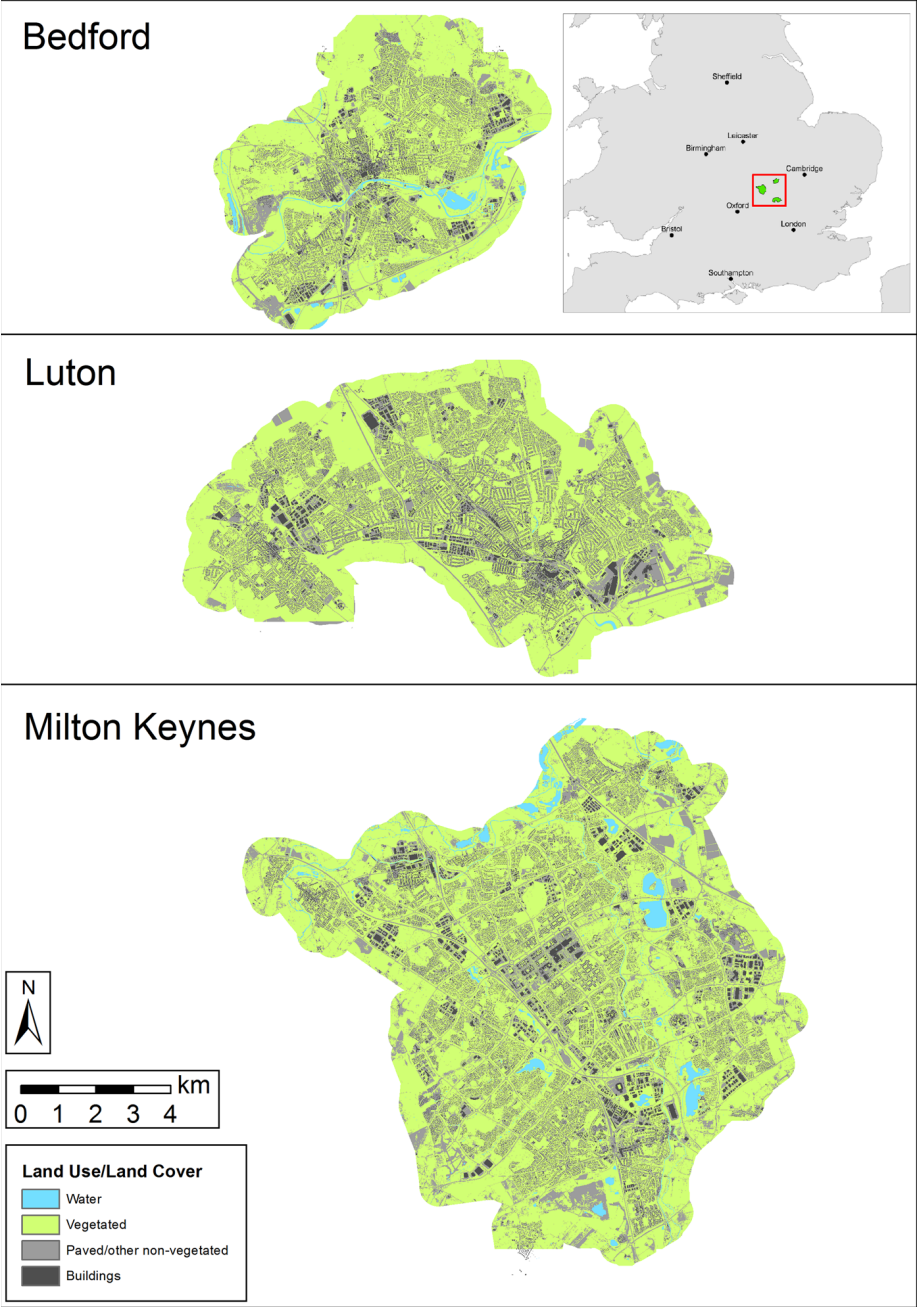
Study area showing locations and land use/land cover classification of Bedford, Luton, and Milton Keynes, UK

The study area for this research combined three English urban landscapes; Milton Keynes, Bedford, and Luton/Dunstable (Fig. 1). Collectively taken as a single study area, the three towns encompass a broad range of urban forms and histories that capture much of the diversity of urban settings within the United Kingdom. Milton Keynes is a planned ‘new town’ developed during the 1960s (52°0′N, 0°47′W), noteworthy for its unique spatial configuration. The town is structured around a grid of major roads designed for speed and ease of automotive travel, rather than the radial pattern common to many more historic English urban landscapes (Peiser and Chang 1999). The town is also characterised by large areas of public green space, possessing many parks and wooded foot and cycle paths (Milton Keynes Council 2015). Milton Keynes’ population in 2011 was 229,941, covering an area of 89 km^2^ (8900 ha) with a population density of 2584 inhabitants per km^2^ (Office for National Statistics 2013).

Bedford (52°8′N, 0°27′W) developed in the Middle Ages as a market centre, and differs to Milton Keynes by possessing both a much longer history and a road network radiating outwards from its centre like many British towns. Its 2011 population was 106,940 and the town covers 36 km^2^ (3600 ha), with a population density of 2971 inhabitants per km^2^ (Office for National Statistics 2013).

Luton (51°52′N, 0°25′W) developed heavily during the nineteenth century as an industrial centre. As such, its urban pattern contains large industrial parks and residential ‘terrace’ housing. Here considered as the combined Luton/Dunstable urban area, the region had a 2011 population of 258,018 and covers 58 km^2^ (5800 ha), with a population density of 4448 inhabitants per km^2^ (Office for National Statistics 2013).

### Land use/land cover and urban form

A 5 m resolution land use/land cover (LULC) dataset was used for analysis, originally classified and resampled from 0.5 m colour aerial photography over the study area obtained from LandMap Spatial Discovery (http://landmap.mimas.ac.uk/) (described in more detail in Grafius et al. 2016). The imagery was taken on 2 June 2009 for Bedford, 30 June 2009 and 24 April 2010 for Luton, and 8 and 15 June 2007 and 2 June 2009 for Milton Keynes, based on cloud-free image availability. Vegetated and non-vegetated surfaces were separated according to a Normalised Difference Vegetation Index (NDVI) threshold. UK Ordnance Survey MasterMap layers were used to distinguish buildings, roads and water bodies.

For the analysis described here, vegetation was treated as a single class in order to focus on broad-scale landscape configuration. Green patch composition at a higher level of detail forms a continuum from mown lawns, through tall meadows, shrubs, hedges and low trees, to the tallest mature trees and woodlands; distinguishing between these vegetation types and treating them as separate classes, while important for understanding ecosystem service provision and patch-scale dynamics, requires detailed and case-specific justifications that were believed to run counter to the landscape-scale objectives of this research. This research thus represents a generalised, high-level approach that distances the analysis from direct applicability to particular species or ecosystem functions but is believed by the authors to have greater potential relevance to urban planners and managers in determining the overall importance of green space location and shape across the urban landscape.

**Fig. 2:**
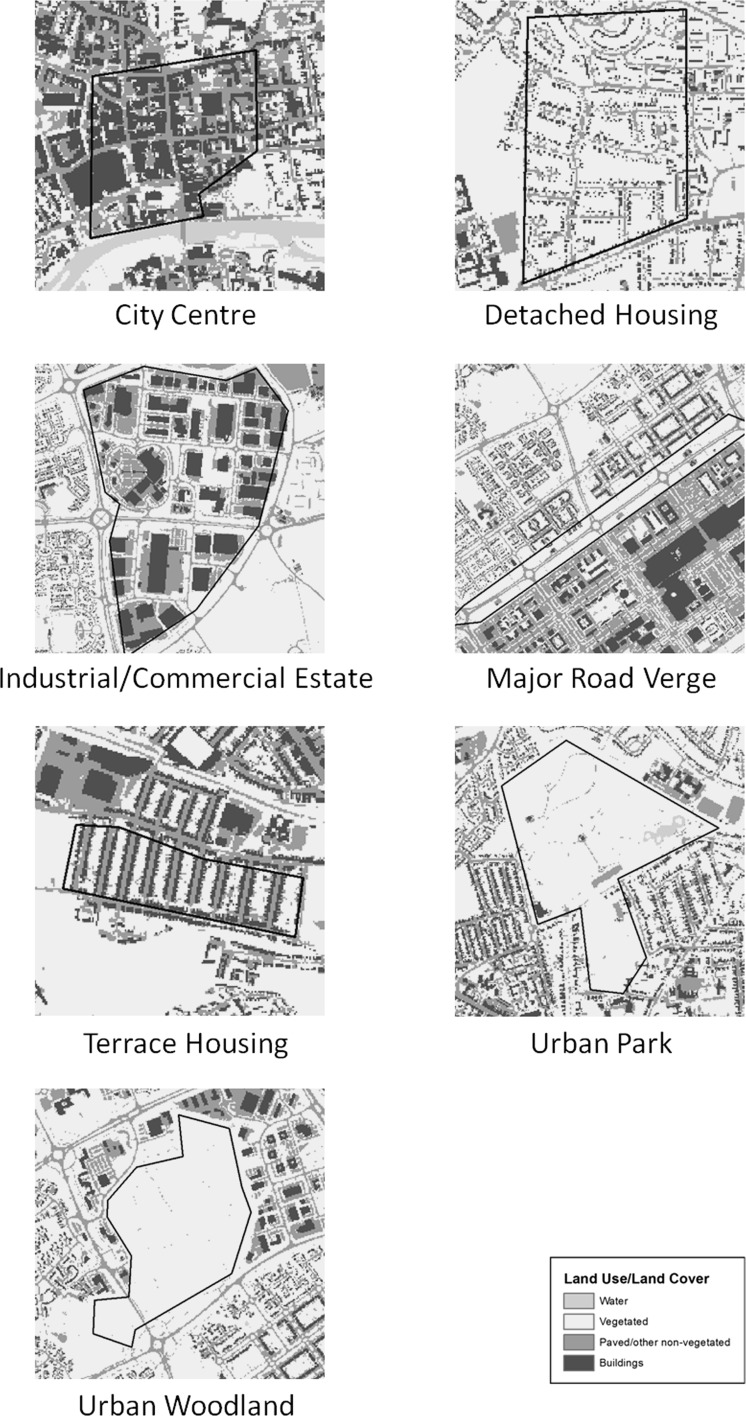
Detailed views showing examples of known urban form types

Seven urban form classes were selected based on known land use/land cover as the major components making up the study area. These were: (1) city centres, dominated by a high density of paved surfaces and buildings with little vegetation cover outside of small, isolated patches; (2) urban parks, relatively large areas of landscaped urban green space containing a mix of grass and trees; (3) urban woodlands, relatively large areas of contiguous woodland surrounded by urban land; (4) row/terraced housing (or ‘townhouses’ outside of Europe), connected medium-density residences exhibiting linear patterns and green spaces in the form of private gardens showing connectedness within blocks but isolation from other patches; (5) single-family/detached residential housing, with more complex road networks than terraced residential and vegetated patches showing increased connectivity; (6) transport corridor verges, involving linear corridors of habitat bordering major roads, river or railways; and (7) industrial and commercial estates, which are heavily paved and dominated by large buildings in similar fashion to city centres but with more regular spacing and little vegetation (Fig. 2).

Three samples of each urban form were visually classified (one from each town) in order to facilitate calculation and comparison of landscape metrics between each type. These sample sites were chosen a priori on the basis of being the largest contiguous and most visually representative examples of each form in the study area. Transport corridor samples all involved major road verges, as river and railway embankments in the study area were generally very narrow and inconsistent in configuration.

### Landscape metrics analysis

A number of landscape metrics were calculated for the urban green spaces within the study area using an 8-cell neighbourhood rule (i.e., inclusive of diagonal as well as horizontal/vertical adjacency), producing tabular outputs (see Online Appendix, Table A1) using Fragstats 4.2 (McGarigal et al. 2012). These metrics were calculated at the patch level (i.e., for each individual green patch) for all vegetated areas in the study area landscape, with non-vegetated areas treated as the background matrix and excluded from analysis. The results within the urban form samples (n = 21) were then compared to assess noteworthy characteristics and differences between urban forms. The patch-level metrics investigated are described below. It should be noted that while Fragstats is capable of producing many additional metrics, those below were chosen a priori in order to represent measures believed to be of greatest interest to researchers and planners for their common use and relative ease of interpretation.

Patch area (AREA) and perimeter (PERIM) were calculated for all vegetated patches. Although sharing a degree of redundancy with other shape indices that derive from them, the inclusion of these simple measurements was deemed relevant to determining their relative impact alongside more complicated shape metrics. Perimeter-Area Ratio (PARA), shape index (SHAPE) and the radius of gyration (GYRATE) measure patch extent and size versus compaction. SHAPE is a simple ratio of perimeter to area, whereas GYRATE is equal to the mean distance between each cell in a patch and the centroid of that patch; as such it is sensitive to patch area. It has a value of zero when patches are single pixels, and increases without limit as patches grow in size.

Fractal dimension index (FRAC) is a measure of fractal shape complexity that returns a value between 1 and 2, where values approaching 1 denote patches with simple perimeters while values approaching 2 indicate greater complexity and convolution (McGarigal 2014). Contiguity (CONTIG) is a metric driven by the occurrence of large, contiguous patches and is reported between 0 and 1. As such, low values in this metric denote small and fragmented patches while numbers approaching 1 represent large contiguous patches (McGarigal 2014; Park et al. 2014).

The core area (CORE) of a patch is defined by a user-supplied edge depth criterion, and represents the area of that patch not impacted by edge effects. Its value can therefore be any number greater than zero, with larger numbers denoting greater variability (McGarigal 2014). The edge depth criterion was chosen as 5 m based on the research of Gallego (2015), which tested core area calculations across the same study area, and determined that 5 m appeared to strike an effective balance across all classes, occurring at the base of the steepest slope when graphed against similar results with other edge depths. Additionally, the number of core areas in each patch (NCORE) and the core area index (CAI) were calculated, the latter being the percentage of core area with respect to total class area (Neel et al. 2004; McGarigal 2014; Wang et al. 2014). This metric may exhibit a measure of redundancy with CORE, but possible differences in the nature of the results informed the decision to investigate this metric.

Euclidean Nearest-Neighbour Distance (ENN) measures proximity between patches of the same class based on the shortest edge-to-edge distance. As such, it acts as a rough proxy for connectivity within the urban form samples.

Several class-level metrics (i.e., summary information pertaining to all patches of a given type) were also calculated to provide additional information, including Largest Patch Index (LPI—percentage of green landscape taken up by largest patch), Core Area Percent of Landscape (CPLAND) and Patch Density (PD—number of patches per 100 ha). Since the analysis contained only a single class (vegetated patches), these metrics produced additional information that could be compared with the means of patch-level data.

All results were summarised via their mean and standard deviation within each urban form, and imported into ESRI ArcGIS Desktop 10.2 and examined spatially for structural patterns and relationships with known urban forms and land uses. This was facilitated by selecting an option in Fragstats to generate a patch ID file with the results.

### Multivariate classification of urban green spaces using landscape metrics

The analysis described above compared landscape metric results with samples of known urban forms within the study area (described previously). However, there was also interest in exploring the ability to characterise different structural types of urban green spaces from landscape metrics without the preconception of known urban forms. To this end, a principal components analysis (PCA) was conducted on the patch-level results above (AREA, PERIM, GYRATE, PARA, SHAPE, FRAC, CONTIG, CORE, NCORE, CAI, and ENN) for the entire study area in order to isolate the multivariate axes that explained the most variation in the results.

Because PCA is a variance reduction technique that collapses input variables into orthogonal multivariate dimensions, it incorporates any and all correlation that is present between input variables and strips it out. Several of the landscape metrics selected for analysis exhibited high correlations between one another (see Supplementary Materials), but the use of PCA outputs rather than direct landscape metric values in subsequent analysis effectively removed the impact of correlation. When studied directly without correction, correlation can have a detrimental effect on analyses (Neel et al. 2004; Wang and Malanson 2007; Cushman et al. 2008; Kupfer 2012; Wang et al. 2014). A degree of correlation between metrics is unavoidable and expected, and although we sought to minimise redundancy between metrics, a higher priority was placed on the inclusion of metrics deemed to be of interest in order to ensure that all relevant aspects of landscape configuration were included in the analysis (cf. Coppedge et al. 2001). The avoidance of correlation between metrics was deemed important but secondary to capturing all relevant aspects of landscape configuration, particularly given the ability of PCA to remove the influence of any correlations present.

The first six principal components resulting from the PCA were subjected to a Ward’s hierarchical cluster analysis to determine where natural groupings occurred in the data with respect to green patch geometry. Ward’s is a minimum variance approach that produces a scree plot, enabling the identification of the optimal number of clusters (Corstanje et al. 2016). For the cluster analysis, the data were standardised by subtracting the column mean and dividing by the column standard deviation. This methodology was adapted from Cushman et al. (2008), but here was focused on the classification of patch types rather than the testing of landscape metrics. PCA and cluster analysis were conducted using JMP software (SAS Institute Inc. 2013). The resulting classification scheme was then compared to known urban forms and modelled ecosystem service provision.

### Comparison with modelled ecosystem service provision

Previous research by the authors (Grafius et al. 2016) used the InVEST 3.1.0 framework (Tallis et al. 2014) to model potential carbon storage, pollination and sediment erosion (treated as the inverse of sediment retention) within the study area described here, based on mapped land cover, published empirical measurements and expert knowledge. This work produced spatially explicit maps of these three ecosystem services, which were then quantitatively compared in ESRI ArcGIS Desktop 10.2 with the urban form samples and the multivariate classification of urban green spaces described above. This enabled the exploration of relationships between potential ecosystem service provision and the structure of the urban landscape, in terms of both urban form and green space structural properties.

## Results

### Statistical results in known urban forms

The means (Table A2) and standard deviations (Table A3) were calculated for all patch- and class-level landscape metrics in each urban form and combined across all three sample regions for each. Some similarities between metric results across different urban form samples (e.g., multiple metrics showing high values in one urban form and low values in another) may be driven by mathematical commonalities between those metrics. However, all tested metrics showed different trends of high and low values from one another in different urban forms, suggesting they all have unique information to contribute.

City centre green patches, generally scattered and small (mean of 0.05 ha), were typified by relatively low mean and standard deviation values for area, perimeter and shape complexity (see Tables A2 and A3). Nearest neighbour distance and patch density were both high but variable. Single-family/detached housing green spaces exhibited low contiguity and core area index given their tendency to exist in narrow corridors around houses and roads. These patches were also highly variable in perimeter, number of core areas, and largest patch index, given the mix of small, isolated patches with larger and more complex patches weaving through private gardens and local parklands. Patches in industrial estates were generally small (mean 0.12 ha) and with little core area and high nearest neighbour distances, similar to city centre patches. They differed from city centre patches by being more variable in patch density, largest patch index and core area percent of landscape. Major road verge patches tended to be long and narrow, exhibiting relatively high values in shape complexity (e.g., mean FRAC value of 1.12), but also high variability (e.g., FRAC standard deviation of 0.12). Terrace/row/townhouse residential green space patches were generally smaller than in detached housing areas with little core area and low variability, except in patch density which varied more highly. Urban parks consisted of large and contiguous green spaces with much core area and low patch density, given how few distinct patches they involved. Variability was relatively high across multiple metrics. Urban woodlands exhibited the largest areas by far, in all tested cases consisting of single large patches. Contiguity and core area was appropriately high to match (means of 0.97 and 21.90 ha, respectively), with generally low variability across numerous metrics given the consistency involved in such large and contiguous patches. Woodland patches also exhibited a high average shape complexity, counter to expectation.

### Multivariate classification of green patch type

The PCA explained 98.4% of the variability present in the landscape metric results with the first six principal components (PC’s; 52.4% by PC1, 24.9% by PC2, 8.8% by PC3, 5.55 by PC4, 5.0% by PC5 and 1.7% by PC6. See supplementary materials for more detail). In the PCA’s scree plot an inflection point was present after the first six components, which were then used for further analysis (after Cushman et al. 2008). Positive and negative Eigenvector loadings for each component represent the factors most strongly influencing them. Component 1 was typified by moderate positive loadings across several metrics (see Table A1 for metric abbreviations and definitions), including AREA, PERIM, GYRATE, SHAPE, CORE, and NCORE; PC 2 was characterised by a high positive loading in PARA and a strong negative loading in CONTIG; PC 3 was driven by a very high loading for ENN; PC 4 included a high loading for FRAC and moderate positive loadings for SHAPE and PARA; PC 5 contained high loadings for GYRATE and CAI; and PC 6 was characterised by a strong negative loading for GYRATE and a positive loading for CAI (see supplementary materials for more detail).

**Fig. 3:**
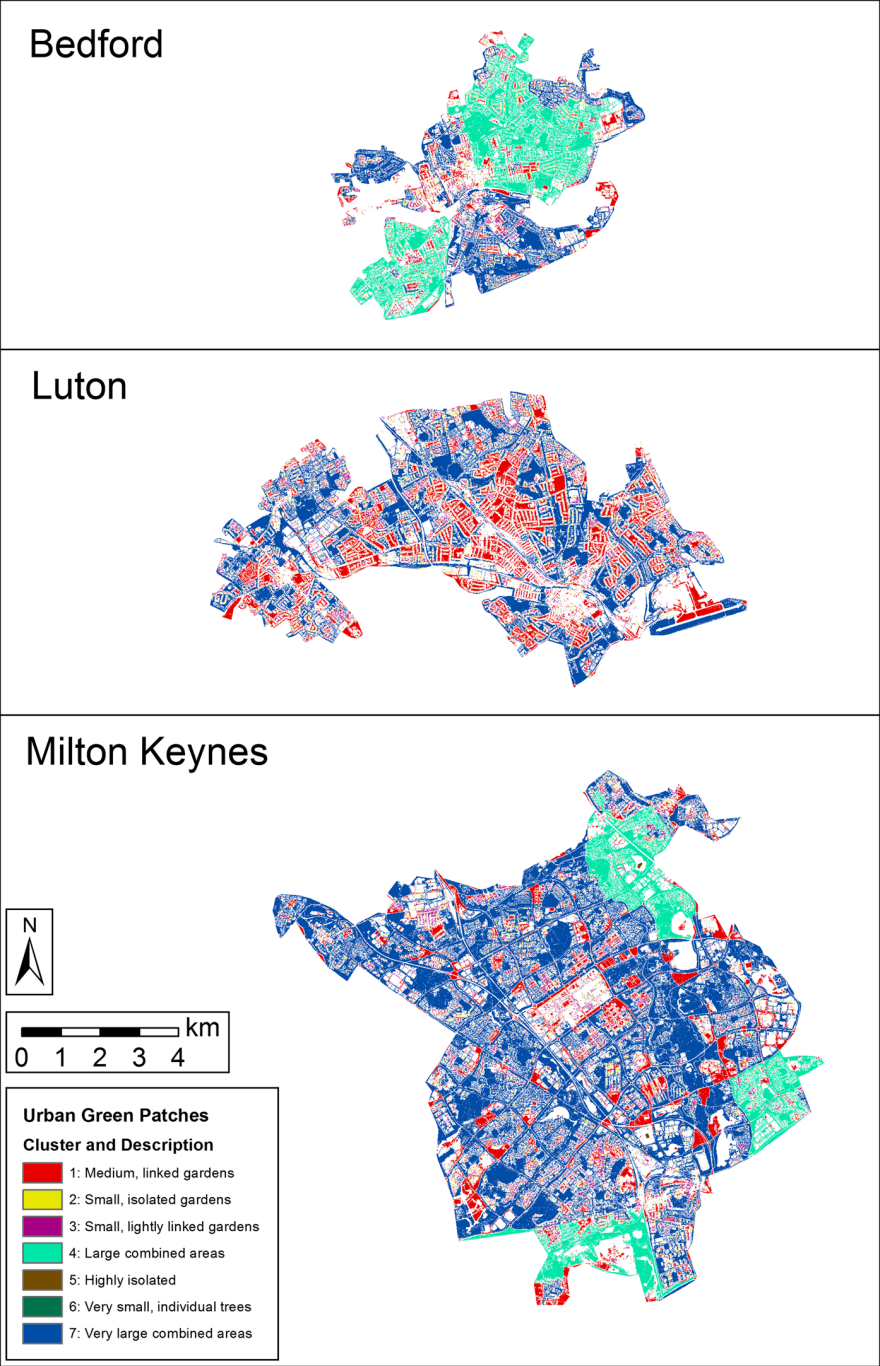
Vegetated patch classification based on principal component analysis and cluster analysis from calculated landscape metrics. White regions of the map are non-vegetated surfaces

Cluster analysis on the first six principal components resulted in a classification of urban green patches that was based on natural groupings in the multivariate space of the landscape metric results (Fig. 3). Seven classes were selected from the cluster analysis based on the location of cluster division points in the dendrogram for cluster membership (see Supplementary Materials). Cluster 1 largely consisted of medium-sized green patches made up of linked residential gardens in areas of terrace or detached housing, otherwise isolated from larger patches. Clusters 2 and 3 both involved small patches; small stands of street trees or individual isolated gardens with complex shapes low on core area, and with Cluster 3 involving some small assemblages of linked gardens. Cluster 4 primarily involved large, complex patches in residential areas. Cluster 5 was visibly typified by high isolation, containing patches unusually far from other green patches. Cluster 6 was comprised of very small patches of only a few pixels, representing very small spaces or individual trees in otherwise un-vegetated areas. Lastly, Cluster 7 contained the largest and most expansive patches, be they large parks and woodlands or highly linked green spaces in residential areas. When considered in the context of the urban form samples, cluster membership is varied but broadly consistent across different samples of the same forms. For example, city centre green patches were predominantly grouped into clusters 1, 2 and 3, whereas detached housing patches more frequently belonged to clusters 1, 2, 3 and 7. Variations between samples appeared to relate to larger landscape differences between towns or regions, as cluster results were driven entirely by the spatial configuration of vegetated patches rather than the human uses that surround them.

**Fig. 4:**
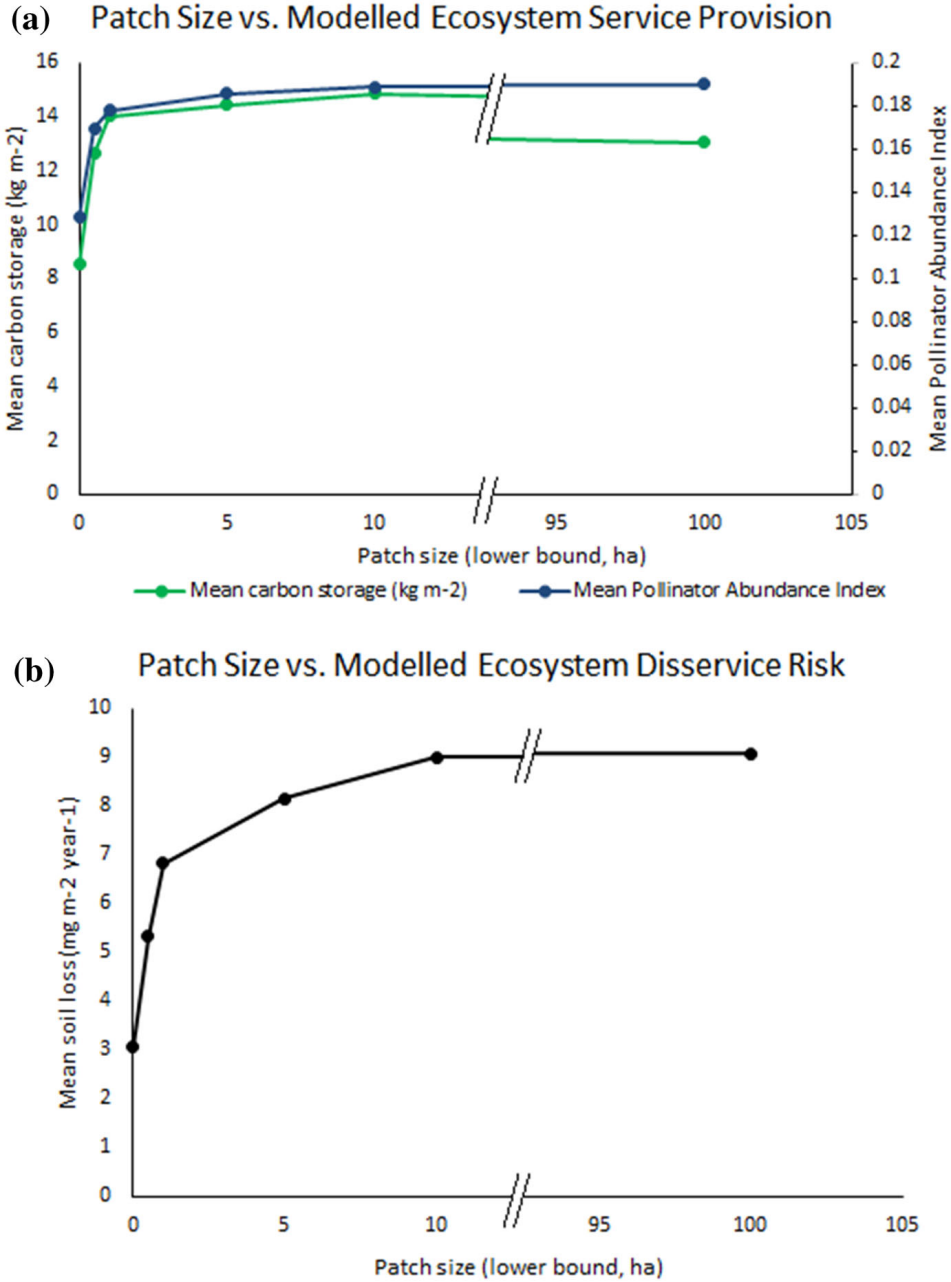
Relationships between green patch size and **a** modelled carbon storage and pollinator abundance index, and **b** modelled mean soil loss. Note that in both cases the X axis has been split to show changes over low patch size values. In Fig. 4a, carbon storage provision drops between 10 and 100 ha relative to pollinator abundance, and the axis split accounts for the visual shift in its line

**Fig. 5:**
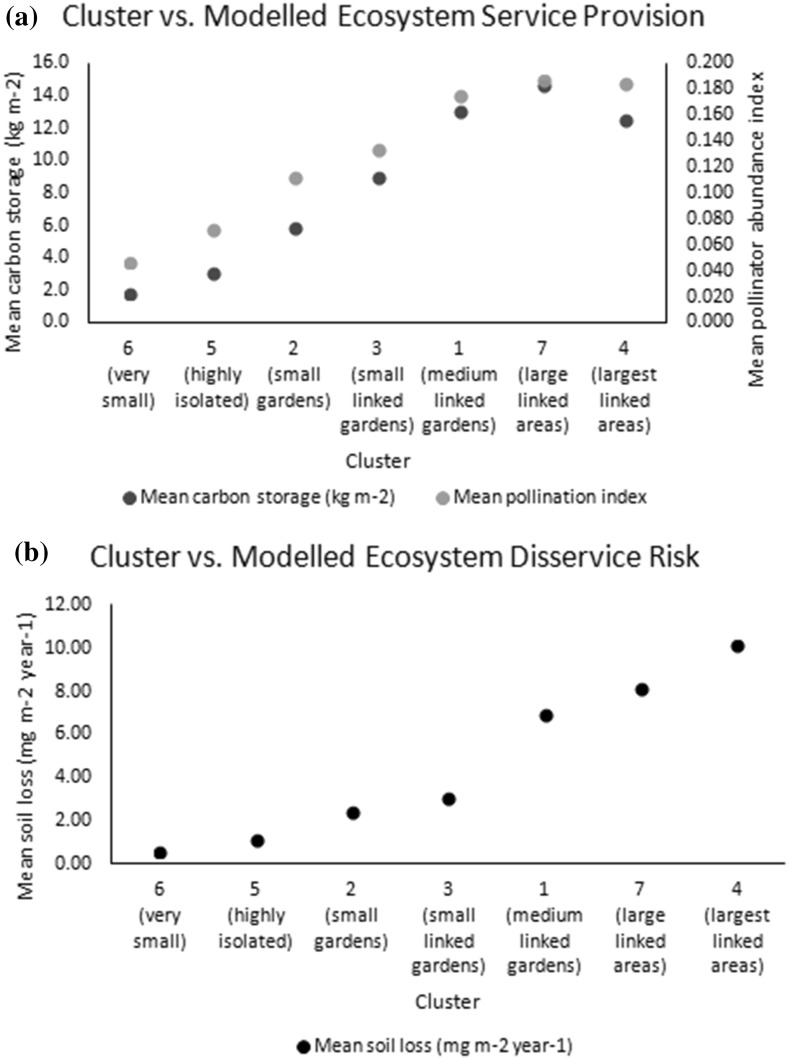
Relationships between cluster (in order of mean patch size) and **a** modelled carbon storage and pollinator abundance index, and **b** modelled mean soil loss

The clusters resulting from this analysis were combined with the results from modelling the provision of three ecosystem services (Grafius et al. 2016). The mean modelled results for carbon storage, sediment loss to erosion (representing the inverse of soil retention as an ecosystem service) and pollinator abundance were calculated across all pixels of each cluster (Table 1). Clusters 1, 4 and 7 (those making up the largest-sized patches) contained the highest mean values for modelled carbon storage and pollinator abundance, but also exhibited the highest modelled risk of soil loss. Clusters 2, 5 and 6 exhibited the lowest modelled risk of soil loss but low values for carbon storage and pollinator abundance. Finally, as cluster membership appeared driven largely by patch area, an additional comparison was made between patch area and ecosystem service provision (Fig. 4). Similar relationships are present; per unit area, carbon storage and pollinator abundance are both higher in larger green patches, as is sediment erosion risk. All three modelled attributes can be seen to increase with green patch size up to a point (approximately 5–10 ha) before levelling off at a seemingly maximum value within the study area. When modelled ecosystem service provision or risk is graphed against the clusters (in order of mean patch size), a similar but more even trend of provision/risk increasing with mean cluster patch size is visible (Fig. 5).

**Table 1:**
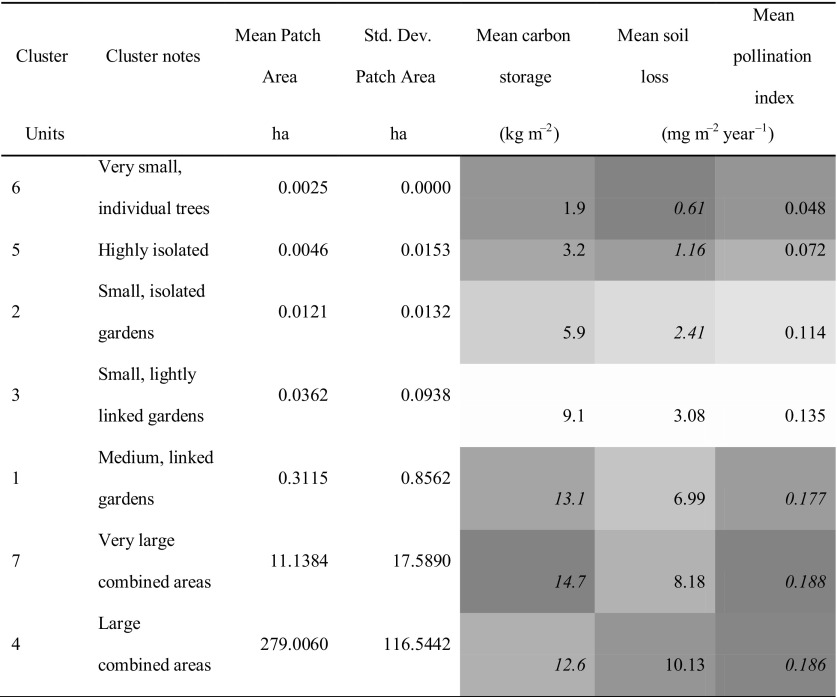
Mean values for modelled carbon storage, soil loss, and pollinator abundance in green space classes resulting from PCA/cluster analysis (clusters ordered by mean patch area)

Blue shading with italicised values highlights relatively high mean values; red shading highlights relatively low values. Note that highlighting scale is inverted for mean soil loss as it represents an ecosystem disservice

Some landscape metrics used in analysis exhibited high correlations with one another (see Supplementary Materials for details). Although this can reduce confidence in the results of multivariate analysis, it was deemed important to the exploratory nature of this research to include all metrics of interest to avoid the loss of measurement of key elements of landscape configuration. Patch area and perimeter correlated strongly with several other metrics; an expected result given that many shape metrics are based on these measurements. Of several tested shape metrics, SHAPE correlated with many of them. Also matching expectation, core area (CORE) and number of distinct core areas (NCORE) correlated highly with one another.

## Discussion and conclusions

The nature of landscape metric calculations can make it difficult to determine which effects are caused by legitimate differences in landscape character and which are artefacts of calculation methods or spurious attributes of landscape geometry. The use of a 5 m spatial resolution in this study, for example, may show different results from a similar analysis conducted at a different scale, and will exhibit a degree of aggregation within the highly-detailed urban matrix. However, a number of calculated differences between urban forms do correspond with expected or explainable qualities, suggesting that landscape metrics analysis on urban green spaces may be useful for exploring how urban form impacts the character of urban green space.

### Landscape analysis of urban form samples

City centres and industrial estates shared a number of similar qualities in their landscape metrics, both containing generally small and isolated green patches. Vegetated areas were generally small, simple and relatively distant from one another, all of which are reflected in multiple metric results. The strongest ability to discern between these forms came from city centres possessing less core area and greater patch density than industrial estates, as the latter occasionally contained larger green patches (e.g., areas of grass lawn) between blocks of artificial surfaces; city centre green patches were consistently very small (reflected in LPI values).

At the opposite end of the development intensity spectrum, urban woodland contains virtually no buildings or paved surfaces, and here consisted of very large and continuous patches. This led to large patch areas and core areas as well as high values in shape complexity metrics that are sensitive to area and perimeter values. Patch density for urban woodland was low, given the few distinct patches involved. Urban parks performed similarly but with less extreme values in many cases, given the reduced sizes and greater variety of shapes involved. Across a number of metrics, urban woodland experienced very low variability relative to urban parkland. Here this will largely be due to the relatively small number of distinct patches in urban woodlands; however, even with larger sample sizes, urban woodlands can be expected to show greater consistency in patch shape and configuration than urban parkland, where greater diversity in design and management is expected.

Housing types and road verges generally occupy the middle of the management intensity spectrum, involving complex mixes of artificial and vegetated surfaces. Among these it was sometimes difficult to discern clear metric differences between urban forms, though green patches in areas of terrace housing were distinctive from detached housing by being smaller and simpler with considerably less core area and low variability in multiple metrics. Green spaces in detached housing, by contrast, were potentially much larger and more spatially connected throughout the sample areas. Detached housing green spaces were also much more variable in shape and size, depending highly on the design of a given residential area. Visually, terrace housing green spaces tend to exhibit a parallel linear alignment caused by the shape of the buildings and road networks; however this characteristic was not represented by any of the tested landscape metrics. Road verges shared similarities with both housing types to an extent that, as an urban form, they may be difficult to distinguish based on landscape metrics alone.

It should be noted that the characteristics revealed here are internal to the urban form samples studied. While forms containing smaller and more isolated green patches (e.g., city centres and industrial estates) will remain more or less self-contained, other forms (e.g., urban parks and major road verges) are likely to contain green patches that cross over into neighbouring urban forms. As such, when analysing an entire urban landscape, green patches in certain forms are unlikely to remain isolated enough to exhibit the characteristics shown here. When urban forms are separated in advance of landscape metrics analysis, green spaces in different urban forms may reveal patterns similar to those revealed here; however when landscape metrics are used to classify an urban landscape whose urban forms have not been previously demarcated, this crossover effect may obscure the classification of some forms such as road verges.

### Landscape characterisation using multivariate analysis

There is value in moving away from preconceived classifications of green space form and instead shaping analysis based on empirical metrics of landscape structure. The methods described here present a way that landscape metrics can be used, through PCA and cluster analysis, to derive a classification scheme based on the observed structure of the landscape and free of the bias that can be potentially introduced by basing the analysis on a pre-existing classification of urban form or land use/land cover. Each landscape has different spatial configurations and ecological dependencies, which confound efforts to apply consistent classifications and approaches between different study areas and confuse the interpretation of individual landscape metrics between research efforts (Cushman et al. 2008; Kupfer 2012). As such, the PCA/Clustering classification method used here (adapted from Cushman et al. 2008) will be unlikely to reproduce the same patch classification from one study area to the next; however it represents a method for addressing these differences and handling the variability of spatial structure in the way most appropriate to the individual landscape of interest.

The clusters resulting from the analysis appeared largely driven by patch area, with some degree of modification based on metrics of shape complexity. When compared to modelled ecosystem service provision, the size of green patches shared a clear positive relationship with carbon storage and pollinator abundance; as modelled, larger patches appear more likely to store more carbon and support more pollinators, not only in an absolute sense but per unit area. These advantages may be somewhat offset by a larger apparent risk of soil loss to erosion, given that they encompass larger vegetated areas away from sealed surfaces. Here, vegetated areas were considered as a single class; categorising them into different classes according to some classification system of vegetation type may provide more revealing results for the provision of particular ecosystem services or certain aspects of ecosystem functioning. Such an approach would depend on the research question being pursued and represent a less generalisable perspective than the broad single-class approach used here.

The extent to which the PCA/Clustering classification method possesses advantages over a simple comparison based on patch size is unclear in this context. Urban landscapes are typified by many small and isolated green patches, so relationships between ecosystem service provision and patch area may be especially strong here. In other landscapes this relationship may be more greatly modified by other factors of landscape structure, such as patch shape complexity and isolation. As such, this approach may represent a useful way to classify and consider landscape structure between different study areas where the same relationships may not be known and cannot be assumed. Patch area is commonly positively associated with connectivity, biodiversity and ecosystem function (Beninde et al. 2015), but other factors can be expected to play a role as well, and the relative contributions of these various factors are likely to vary from one landscape to the next. Further, challenges persist around the sensitivity of landscape metrics to pixel resolution/grain size (Wickham and Riitters 1995; Zhu et al. 2006) and the high level of redundancy between many metrics (Riitters et al. 1995; Cain et al. 1997). A multivariate approach enables researchers to bypass some of these issues in order to consider many possible factors and explore which ones encompass a majority of the variability in a landscape, and by extension possibly the factors that most directly impact ecosystem function. Issues pertaining to the downscaling and upscaling of landscape metrics as functional and structural landscape indicators nevertheless remain a challenge that must be carefully considered (Uuemaa et al. 2005; Grafius et al. 2016). Our analysis was conducted at a spatial resolution of 5 m, which was deemed appropriate based on the ability to adequately represent landscape features of interest (e.g., small stands of trees and sizeable individual ones) while remaining computationally feasible (Grafius et al. 2016). The optimal spatial resolution for a given analysis depends on this ability to capture relevant landscape features; the appropriate spatial resolution is therefore that which adequately captures the smallest landscape features deemed to be important to the research question. Any features too small to be represented should be considered unimportant, otherwise finer-scale data should be used if at all possible to avoid missing key relationships and patterns.

Correlation between landscape metrics was allowed in this analysis in order to include the measurement of important aspects of landscape configuration and explore the characteristics and utility of commonly-used patch-level metrics. Additionally, the testing of correlation between metrics highlights which metrics should perhaps be included or excluded in similar future studies (see Supplementary Materials). Patch area and perimeter in particular were included here in order to assess their relationships with urban green space configuration and other metrics, but as simple measurements from which other, more complicated shape metrics derive, would commonly not be included in more rigorous analysis prioritising the avoidance of correlation. Shape index (SHAPE) and radius of gyration (GYRATE) would also appear, based on their high redundancy with other metrics (r^2^ > 0.5 in some cases), to be metrics that should be considered for exclusion from such analyses, given the lower correlations exhibited by similar shape metrics such as FRAC and CONTIG. Future studies taking a more rigorous perspective on metric selection can benefit from research specifically aimed at testing their correlation and sensitivities (e.g., Cushman et al. 2008; Wang et al. 2014).

Graphing modelled carbon storage, pollinator abundance, and potential soil erosion against patch size revealed clear trends. In all three cases, service provision was relatively lower in small green spaces, but quickly increased with patch area before levelling off at a maximum value. All three modelled attributes showed very similar relationships. This result strongly suggests that carbon storage and pollinator abundance share a positive relationship with green patch area in urban settings; a relationship which may extend to other ecosystem services and characteristics not modelled here such as human wellbeing (Cox et al. 2017b), biodiversity and connectivity (Grafius et al. 2017). Potential soil erosion also increased with patch area due to the decreased presence of paved surfaces, suggesting some trade-off and risk. If the benefits from large green patches in increased ecosystem service provision can be seen to outweigh the risks from disservices such as potential erosion, this would have powerful implications for urban design and support the importance of well-connected green infrastructure in creating healthy cities. Further, the nature of the relationships suggests that patch sizes around 10 ha in area may maximise the areal density of carbon storage and pollinator abundance, with larger patches providing no additional benefit per unit of ground area (Fig. 4).

Concurrent to this, graphing modelled ecosystem service provision and risk against the clusters generated by multivariate analysis of landscape metrics showed a similar trend of increasing provision and risk with increasing patch size. Unlike when graphing these directly against patch size, the trends are smoother and the relationships clearer when using clusters, as the cluster generation process is driven by the characteristics of the data, leading to more natural divisions in patch size as well as seeing modification by other landscape factors which may also impact ecosystem service provision. Although the relationship is not identical, the advantages of increasing patch size appear to stabilise after a maximum size of around 10 ha (cluster 7 in Fig. 5) in the cluster comparison as well as the direct patch size comparison; whereas the results suggest that the risk of soil erosion may continue to increase with larger patch sizes. It is interesting to note that this size meshes with findings that 10 ha may act as a minimum threshold size for effectively supporting urban bird species richness (Nielsen et al. 2014), lending further support to the notion that planners can maximise urban environmental benefits by aiming to create green spaces around this size.

The approach explored here offers value to landscape ecology research seeking to circumvent some of the challenges and shortcomings of calculating and interpreting landscape metrics. The findings of this research also have relevance to green infrastructure planning in urban systems. That larger, more contiguous green patches tend to support ecosystem functioning and services more effectively than small or complex patches low on core area is not a new finding (Saunders et al. 1991; LaPoint et al. 2015). Of particular relevance however is the suggestion by these results that a patch area of approximately 10 ha may approach the optimal size for urban green spaces when the provision of certain ecosystem services is of primary interest. Although patch size is far from the only consideration in urban green infrastructure design, planning efforts informed by this may contribute to urban ecological health and ecosystem service provision.

### Conclusions

Using landscape analysis on the green spaces of different urban forms, it is possible to highlight measures of size, shape and configuration that discern different types of green spaces from one another. The ability to so characterise urban green space form provides a framework that can be used to quantify the properties of these green spaces, and in turn compare and correlate them to other features of the urban landscape such as ecosystem service provision. In doing so, relationships can be drawn between ecosystem services and the structure of the urban landscape in ways that can inform sustainable urban planning and design practices. As studied here, larger urban green spaces appeared to facilitate a greater provisioning per area of carbon storage and pollinator abundance, while also carrying higher risks of soil erosion; however this relationship reached its maximum around 10 ha of patch area. The application of methods similar to those used here in other cities and landscape types could prove insightful and further the ability to discern ecological value, both in terms of function and service provision, from landscape metrics analysis, as well as highlighting important structural drivers of ecosystem function unique to each landscape.

## Electronic supplementary material

Below is the link to the electronic supplementary material.

Supplementary material 1 (DOCX 85 kb)

Supplementary material 2 (DOCX 21 kb)
