# Supplementary material for: Linking ecosystem services, urban form and green space configuration using multivariate landscape metric analysis
Source: Landsc Ecol. 2018 Feb 19;33(4):557–73. doi: 10.1007/s10980-018-0618-z (PMC6561088; doi:10.1007/s10980-018-0618-z)
Supplement: Supplementary file 1 — Supplementary material 1 (DOCX 85 kb) [file 10980_2018_618_MOESM1_ESM.docx]

## Linking Ecosystem Services, Urban Form and Green Space Configuration Using Multivariate Landscape Metric Analysis

Darren R. Grafius, Ron Corstanje, and Jim A. Harris

# Supplementary Materials

*Table S1: Eigenvectors from Principal Component Analysis (PCA) showing strength of influence of each landscape metric for first six principal components. These show the extent to which each component was influenced by the different factors that were used in the PCA, and in turn how they contribute as combined components to the cluster analysis.*

|  | **Prin1** | **Prin2** | **Prin3** | **Prin4** | **Prin5** | **Prin6** |
| --- | --- | --- | --- | --- | --- | --- |
| **AREA** | 0.35970 | 0.28526 | -0.03864 | -0.11529 | -0.12040 | 0.08635 |
| **PERIM** | 0.35982 | 0.28168 | -0.04397 | -0.08157 | -0.16156 | 0.15431 |
| **GYRATE** | 0.37533 | -0.02446 | 0.07479 | 0.15521 | 0.40161 | -0.57905 |
| **PARA** | -0.20810 | 0.45815 | -0.01144 | 0.39357 | 0.33746 | 0.27478 |
| **SHAPE** | 0.37908 | -0.06718 | 0.04598 | 0.38096 | 0.18740 | -0.26791 |
| **FRAC** | 0.20977 | -0.36276 | -0.00033 | 0.70200 | -0.30973 | 0.36856 |
| **CONTIG** | 0.22487 | -0.47424 | 0.03189 | -0.26740 | -0.27711 | -0.05194 |
| **CORE** | 0.35382 | 0.28093 | -0.03458 | -0.12708 | -0.09327 | 0.04130 |
| **NCORE** | 0.35920 | 0.26449 | -0.04075 | -0.06975 | -0.16159 | 0.19378 |
| **CAI** | 0.24966 | -0.31850 | 0.18738 | -0.26506 | 0.64180 | 0.55477 |
| **ENN** | -0.04635 | 0.13210 | 0.97455 | 0.01902 | -0.17227 | -0.02459 |

*Table S2: Correlation coefficients for paired results of landscape metrics used in multivariate analysis. Colour coded to show strong positive and negative correlations. Some metrics were included in analysis despite high correlation values due to the importance of capturing the aspects of landscape configuration that they measure.*

|  | **AREA** | **PERIM** | **GYRATE** | **PARA** | **SHAPE** | **FRAC** | **CONTIG** | **CORE** | **NCORE** | **CAI** | **ENN** |
| --- | --- | --- | --- | --- | --- | --- | --- | --- | --- | --- | --- |
| **AREA** | - |  |  |  |  |  |  |  |  |  |  |
| **PERIM** | 0.9778 | - |  |  |  |  |  |  |  |  |  |
| **GYRATE** | 0.7169 | 0.6918 | - |  |  |  |  |  |  |  |  |
| **PARA** | -0.1166 | -0.1210 | -0.3961 | - |  |  |  |  |  |  |  |
| **SHAPE** | 0.6744 | 0.7002 | 0.9121 | -0.4313 | - |  |  |  |  |  |  |
| **FRAC** | 0.1369 | 0.1521 | 0.4459 | -0.5781 | 0.6212 | - |  |  |  |  |  |
| **CONTIG** | 0.1307 | 0.1353 | 0.4399 | -0.9815 | 0.4919 | 0.6739 | - |  |  |  |  |
| **CORE** | 0.9937 | 0.9483 | 0.7205 | -0.1132 | 0.6522 | 0.1289 | 0.1272 | - |  |  |  |
| **NCORE** | 0.9518 | 0.9910 | 0.6825 | -0.1388 | 0.7158 | 0.1687 | 0.1547 | 0.9134 | - |  |  |
| **CAI** | 0.2466 | 0.2347 | 0.6350 | -0.6174 | 0.5910 | 0.4333 | 0.6823 | 0.2501 | 0.2510 | - |  |
| **ENN** | -0.0195 | -0.0219 | -0.0725 | 0.1818 | -0.0945 | -0.1517 | -0.1781 | -0.0182 | -0.0250 | -0.0717 | - |

a)

Eigenvalue

b)

c)
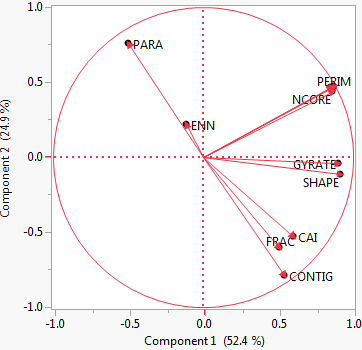


*Figure S1: Scree plot (a), Eigenvalue/principal component cumulative percentage table (b), and PCA loadings graph for principal components 1 and 2 (c) from the Principal Component Analysis. The first six principal components were used in analysis based on cumulative percentages of explained variability and the scree plot's inflection point.*

*
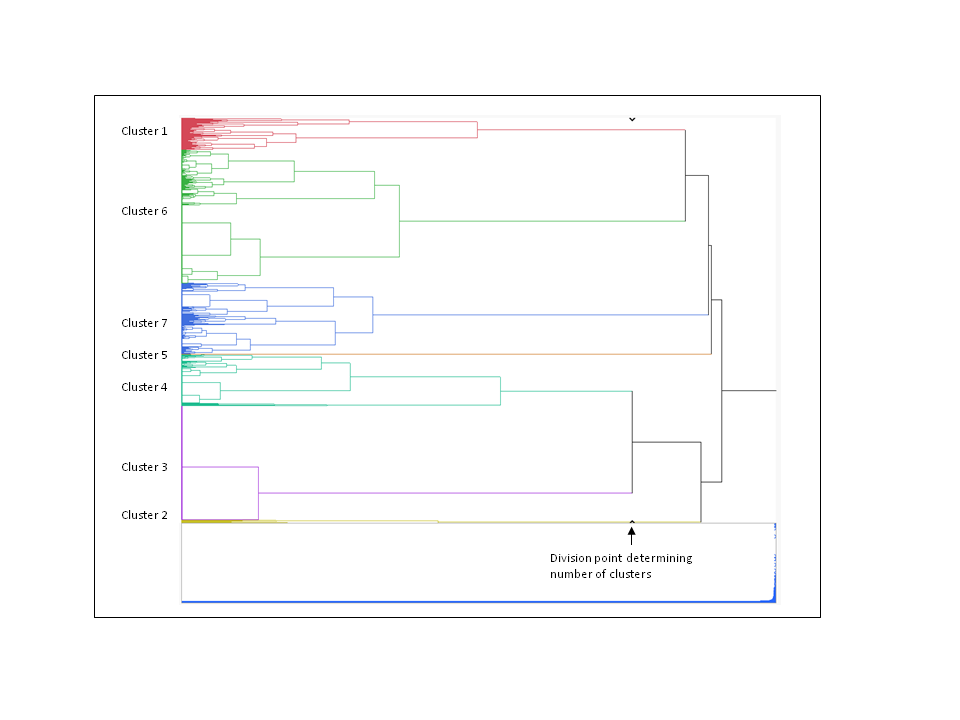
*

*Figure S2: Dendrogram from Ward's Hierarchical Cluster Analysis. Seven clusters were used for analysis based on cluster division points. Each colour in the dendrogram represents one of the clusters resulting from the analysis (corresponding to colours used in main text Figure 3), which were then mapped as each data point was associated with a patch on the landscape. The black arrows denote the division point used to determine the number of clusters.*
