# Supplementary material for: Linking ecosystem services, urban form and green space configuration using multivariate landscape metric analysis
Source: Landsc Ecol. 2018 Feb 19;33(4):557–73. doi: 10.1007/s10980-018-0618-z (PMC6561088; doi:10.1007/s10980-018-0618-z)
Supplement: Supplementary file 2 — Supplementary material 2 (DOCX 21 kb) [file 10980_2018_618_MOESM2_ESM.docx]

## Linking Ecosystem Services, Urban Form and Green Space Configuration Using Multivariate Landscape Metric Analysis

Darren R. Grafius, Ron Corstanje, and Jim A. Harris

**Appendix - Tables**

*Table A1: Landscape metrics used in analysis with acronyms, units (where applicable) and notes on interpretation. Metrics are patch level unless otherwise noted.*

| **Acronym** | **Metric (units)** | **Notes** |
| --- | --- | --- |
| AREA | Patch area (ha) |  |
| PERIM | Patch perimeter (m) | Indicative of shape complexity but highly sensitive to patch size. |
| PARA | Perimeter-Area Ratio | Simple ratio of the length of a patch’s perimeter to its area. |
| GYRATE | Radius of Gyration (m) | Mean distance between patch cells and patch centroid. Sensitive to patch area. |
| SHAPE | Shape index | A metric of shape complexity that aims for simplicity while adjusting for patch size sensitivity. |
| FRAC | Fractal dimension index | Another metric of shape complexity that reduces sensitivity to patch size and scale. |
| CONTIG | Contiguity index | Favours large, contiguous patches. |
| CORE | Core area (ha) | Area unaffected by edges, defined here as area <5m from patch edge. |
| NCORE | Number of core areas | Indication of shape complexity. |
| CAI | Core area index (%) | Patch core area divided by total patch area. |
| ENN | Euclidean nearest-neighbour distance (m) | Distance to nearest patch of the same type. |
| PD | Patch density | Class metric - Number of patches per 100 ha. |
| LPI | Largest patch index (%) | Class metric - Area of largest patch divided by total class area. |
| CPLAND | Core area percentage of landscape (%) | Class metric |

*Table A2: Mean values for tested landscape metrics of green space patches in seven sampled urban forms. Blue shading with italicised values highlights relatively high mean values in a given metric for each urban form; red shading highlights relatively low values. ENN is stated as n/a for urban woodland because each sample consisted of a single woodland patch.*

| **Urban Form** | **AREA** | **PERIM** | **GYRATE** | **SHAPE** | **FRAC** | **CONTIG** | **CORE** | **NCORE** | **CAI** | **ENN** | **PD** | **LPI** | **CPLAND** |
| --- | --- | --- | --- | --- | --- | --- | --- | --- | --- | --- | --- | --- | --- |
| *Units* | *ha* | *m* | *m* | *n/a* | *n/a* | *n/a* | *ha* | *n/a* | *%* | *m* | *Patches/100 ha* | *%* | *%* |
| City Centre | 0.05 | 139.70 | 7.64 | 1.48 | *1.12* | 0.27 | 0.01 | 0.43 | 3.34 | *12.71* | *2457.87* | 16.90 | 25.05 |
| Detached Housing | 0.31 | 543.50 | 9.25 | 1.47 | 1.09 | 0.27 | 0.15 | *1.68* | 3.55 | 10.63 | 385.01 | *69.78* | 44.48 |
| Industrial Estate | 0.12 | 226.88 | 9.27 | 1.46 | 1.11 | 0.26 | 0.05 | 0.71 | 3.57 | *12.62* | *1319.80* | 46.33 | 36.80 |
| Major Road Verges | 0.58 | 622.00 | *33.10* | *1.73* | *1.12* | 0.28 | 0.39 | 0.78 | *13.21* | 11.19 | 177.37 | 40.90 | *65.94* |
| Terrace Housing | 0.07 | 185.41 | 9.40 | 1.47 | 1.10 | 0.29 | 0.02 | 0.87 | 5.86 | *11.71* | *1491.20* | 17.41 | 30.90 |
| Urban Park | *5.54* | *1934.74* | *55.20* | *1.85* | 1.09 | *0.45* | *4.82* | *2.32* | *28.50* | 11.46 | 18.54 | *95.63* | *86.04* |
| Urban Woodland | *23.42* | *4130.00* | *239.57* | *2.22* | *1.13* | *0.97* | *21.90* | *1.67* | *92.70* | n/a | 5.09 | *100.00* | *92.70* |

*Table A3: Standard deviations for tested landscape metrics of green space patches in seven sampled urban forms. Blue shading with italicised values highlights relatively high standard deviations in a given metric for each urban form; red shading highlights relatively low values. Note that PD, LPI and CPLAND are class metrics and thus variability is only calculated across n=3 samples. ENN is stated as n/a for urban woodland because each sample consisted of a single woodland patch.*

| **Urban Form** | **AREA** | **PERIM** | **GYRATE** | **SHAPE** | **FRAC** | **CONTIG** | **CORE** | **NCORE** | **CAI** | **ENN** | **PD** | **LPI** | **CPLAND** |
| --- | --- | --- | --- | --- | --- | --- | --- | --- | --- | --- | --- | --- | --- |
| *Units* | *ha* | *m* | *m* | *n/a* | *n/a* | *n/a* | *ha* | *n/a* | *%* | *m* | *Patches/100 ha* | *%* | *%* |
| City Centre | 0.25 | 605.55 | 11.29 | 0.91 | *0.11* | 0.21 | 0.09 | 2.18 | 9.50 | *4.66* | *507.89* | 6.00 | 2.07 |
| Detached Housing | *3.71* | *6071.25* | 30.84 | *2.10* | 0.10 | 0.20 | 1.75 | *20.33* | 11.00 | 1.41 | 281.62 | *33.25* | 6.38 |
| Industrial Estate | 1.38 | 1747.18 | 21.30 | 1.13 | *0.11* | 0.21 | 0.75 | 5.16 | 10.06 | *5.78* | *1082.28* | *29.86* | *15.94* |
| Major Road Verges | 2.15 | 2036.20 | *90.23* | *1.48* | *0.12* | *0.31* | 1.53 | 2.26 | *25.48* | 1.62 | 202.39 | 3.81 | 5.32 |
| Terrace Housing | 0.31 | 680.16 | 15.02 | 1.00 | 0.10 | 0.23 | 0.10 | 3.69 | 12.50 | *3.06* | *974.96* | *16.32* | *9.10* |
| Urban Park | *13.11* | *4080.35* | *106.72* | *1.50* | 0.10 | *0.38* | *11.71* | 4.76 | *34.86* | 1.72 | 2.53 | 6.68 | 5.17 |
| Urban Woodland | *13.22* | 872.75 | 16.93 | 0.33 | 0.03 | 0.01 | *12.89* | 1.15 | 2.88 | n/a | 2.18 | 0.00 | 2.88 |
